# Albuminuria in Pregnancy and Its Relation to Puerperal Convulsions

**Published:** 1872-06

**Authors:** Geo. T. Campbell

**Affiliations:** Skaneateles, N. Y.


BUFFALO

### Medical and Surgical Journal.

VOL XI. JUNE, 1872. No. 11

### Original Communications.

:o:

ART. I.—*Albuminuria in Pregnancy and its relation to puerperal Convulsions.* By GEO. T. CAMPBELL, M. D. Skaneateles, N. Y.

Read before the Central New York Medical Association.

Mr. President and Gentlemen;—

I will first read the report of a few cases taken from my note book, as an introduction to the paper I offer you to day.

Case No. 1.—Was called early in the morning of Sept. 16th, 1868 to visit Mrs. J. H. in the town of Sennett, aged 30, Primipara, considered herself about eight months along in her pregnancy. Was taken with labor pains during the night while visiting at the house of a relative.

Previous to my arrival she had had two convulsions. Found her much excited, with occasional twitching of the facial muscles, rather pale, much thiner than before her pregnancy, no œdema of extremeties. Urine contained a moderate amount of albumen. Os Uteri fully dilated. Head of child low down in the inferior strait, occiput under the arch of the pubis. Had had no pain for an hour, applied forceps and delivered her of a living male child. Placing my hand on the abdomen found that the uterus contained another fœtus, gave her a drachm of fl. ex. ergot and waited, in the mean time chloroform had been moderately administered, whenever the convulsive muscular movements come on. In about 30 minutes after the delivery of the first child a characteristic convulsion occurred lasting about 60 seconds. As no labor pain had been produced by the ergot, soon as the convulsion ceased and before she recovered her consciousness, I introduced my hand, ruptured the membranes and delivered her of another living child, by podalic version. Firm compression being applied to the abdomen, I repeated the ergot, but the drug seemed to produce no effect. The uterus did not contract down well, and soon I discovered that internal hemorrhage was going on. I again introduced my hand into the uterus, the patient at this time being partly conscious resisted, but by holding her hands and feet I succeeded in bringing on uterine contractions, which expelled the placenta with quite a large quantity of blood. No more hemorrhage occurred. After the patient had regained her consciousness an opiate was given. No more convulsions. Next day mother and babies were doing well. The specimen of urine saved was lost by breaking of the vial. The next day there was no albumen.

Case No. 2.—Mrs. E. of Spafferd, Aged 21. Medium height, fair complexion, primipara. In the 8th month of her pregnancy, was taken with convulsions about 3 o'clock in the morning of March 14th, 1870. Dr. Tripp of Borodino was called to attend her. He opened a vein in the arm, but said, did not get much blood, gave a cathartic and used chloroform. I saw her in consultation about 3 o'clock P. M. At this time she was deeply comatose, face pale and puffy, hands and feet œdematous, respiration 36, heavy and labored, pulse 150. The urine had been scanty and highly colored for two weeks, the day before the attack she had an intense headache. The urine was drawn and found high colored, with a specific gravity of 1030; by heat it becomes solid with albumen. She had had already thirteen convulsions. No labor pains had been observed. On examination the os uteri was open sufficient to admit the end of two fingers, vertex presenting O. A. L. No beating of fœtal heart could be detected.

The case seemed nearly desperate and we concluded that the sooner the uterus could be emptied of its contents, the better would be her chances for recovery. It was very evident that there was no immediate prospect of this occurring by the natural process and we felt that if we waited for this to take place, there would be great danger of her dying undelivered. So we concluded to deliver her as soon as the os could be dilated sufficient to operate, accordingly the smallest size of Barnes' Dilators was introduced and injected full by the pump, this was very soon expelled into the vagina. I then inserted and filled the largest size, which was also discharged in a short time. The hand was then introduced into the vagina, and was soon able to dilate the os sufficient to pass it into the uterus. Ruptured the membranes and felt for the cord; there was no pulsation, seized the feet, turned, and delivered her of a dead child. The placenta soon followed with but moderate hemorrhage.

Soon after placing the patient in a clean bed, another convulsion came on, applied cold to the head and heat to the extremeties. Should have given Croton Oil, but we were in the country about six miles, and neither of us had any with us, gave Rochell Salts, Chloroform to be continued same as before. I then left. Dr. Tripp to remain. I learned afterwards that the salts operated very thoroughly early in the evening. The convulsions continued through the night coming on from half an hour to an hour apart. They were kept in check somewhat by the chloroform. After 9 o'clock in the morning of the 15th, no more occurred, at noon was aroused sufficient to take a cup of beef tea, first regained her consciousness on the evening of the 16th. Being in the neighborhood about two weeks after I called to see her, found her sitting up, bright and cheerful. She had just been told that day, the condition she had been in. So profound had been the impression on the brain she had forgotten all that had transpired for two weeks previous to her sickness, and cannot recall any of the events occurring during that time, to this day.

Case No. 3.—Mrs. T. B., Multipara consulted me about the middle of May 1870. She was in her 4th month of pregnancy. There was puffing of hands and face, feet swollen so as to require the largest size of men's slippers to wear. Urine small in quantity and contained albumen, could find no casts under the microscope. Ordered saline cathartics and diuretes, vegetable diet, to avoid tight lacing, and to let me know if no improvement took place or if any unpleasant symptoms should arise. Heard nothing more until the 10th of July, she said that two or three weeks before the œdema rapidly disappeared, with a considerable of urinary discharge, that her breasts also shrank down and lost their fullness; since that time had felt no motion. On examination, the urine contained no albumen. Told her that probably the child was dead and that she would likely miscarry.

July 30th, the membranes ruptured while at work, about 3 o'clock P. M. Labor pains supervened and she was delivered in the evening of a fœtus of about 6 months growth. It had been some time dead. The funis just as it entered the umbilicus was contracted to about the size of a large knitting needle cutting off the circulation and was the probable cause of the death of the fœtus. The point of interest in this case was the disappearence of the œdema and cessation of the secretion of albumen with the urine on the death of the child, some weeks before it was expelled from the uterus.

Case No. 4.—Mrs. K. was seen March 29th 1868 in consultation with Dr. Bartlett. The patient was six months pregnant, was complaining of severe headache, vomiting up every thing taken into the stomach, mind rather sluggish, general œdema, urine scanty and solid with albumen, on testing by heat and nitric acid. The treatment was saline in the form of Sedlitz powders until a full and free evacuation of the bowels should take place. Chloroform to the epigastrium, non-nitrogenous diet and hot air baths. I learned from Dr. B. that the vomiting ceased after the first powder was given, that he continued them until there were repeated watery discharges from the bowels, that the result of the treatment adopted was favorable. The œdema became less, the headache disappeared, the urine became more abundant.

April 1st, labor pains came on and she was delivered of a still born child.

April 3d, Urine free from Albumen. Patient comfortable.

April 5th, Owing to some error in the diet, vomiting again came on and the urine was again loaded with albumen, same treatment as before.

April 8th. Urine free from Albumen and patient did well from this time.

Case No. 5.—Mrs. R., Daughter of one of the oldest members of the Onondaga Co. Med. Society was taken in labor May 27th, 1868 in the forenoon. Had two convulsions before delivery which occurred soon after-noon without artificial interference, but the eclamptic attacks continued from half an hour to an hour and a half apart. About 10 o'clock in the evening Dr. Bartlett and myself were added to the consultation. The father, Dr. Porter and Dr. Earll, had been with her during the day. At this time she was profoundly comatose, the breathing stertorous and so loud that I heard it while entering the gate some rods from the house, the window being open. On drawing the urine it was found loaded with albumen. The convulsions continued through the night, until 6 o'clock the next morning when they ceased, but the coma continued without any muscular movements except the involuntary ones of breathing and circulation. She died that evening.

These cases occurring under my own observation within a little more than two years led me to choose the subject of albuminuria in pregnancy, and its relation to puerperal convulsions, as the subject of the paper I present for your consideration to day. And, although I may not be able to give you anything new in the history or management, I am sure you will all agree that there is no danger of being too often reminded of all that is known in regard to one of the most grave and fearful incidents of the lying in chamber. One where so much depends on the prompt and intelligent action of the medical attendant. There may be no time to consult authorities or call in counsel. And where two lives, mother and child, the central lights of a home, might be sacrificed, by the hesitation or ignorance of him to whose care they had been intrusted.

The literature of the relation of uræmia to eclampsia puerperalis extends back only about twenty years, previous to that time uræmic intoxication was never mentioned by writers or teachers as a cause of this malady. Nervous irritation, congestion and moral influences were regarded as the sole cause, and were considered sufficient to account for all the phenomena connected with it.

In about the years 1850 & 51 the fact of the coincidence of puerperal eclampsia and albuminuria, was verified and established by such men as Simpson Devilliers, Regnault Cazeaux, Cormack Blot, Frericks, Lintzman, Carl Brown and many othess and a new pathway was opened in this important and interesting field of medical research.

Since that time no branch of obstetrical science or practice has been more thoroughly discussed and explored than this. All the appliances of modern art, and the skill of modern culture, have been employed in the search for the truth concerning renal complications and uræmic poisoning in pregnant woman. And there is nothing in all that pertains to the practice of medicine, that more invites investigation, more fully awakens our deepest sympathies, or has better paid in results, for the time and labor spent than this. Statistics show a diminution of the rate of mortality of about 50 per cent.

As to the causes of albuminuria in pregnancy, there are various opinions. There is no doubt however, but that a certain proportion are from acute Bright's disease, some of which may come from the ordinary causes of that complaint, exposure to cold, bad food, abuse of alcohol &c., but mostly from the effects of the pregnancy itself, for we know it to be a fact that pregnancy like scarlatina and some other diseases, may be an exciting cause of a temporary Bright's disease. The theory that this morbid condition is caused by pressure of the gravid uterus on the renal veins, thus producing congestion of the kidneys, has several elements of plausibility. The fact that a large majority of the cases of eclampsia are primipara, and that twin pregnancies are more prone to it than single ones, has been regarded as presumptive proof of the truth of this doctrine, by a large class of able practitioners.

The unyielding abdominal walls of the one, and the size of the uterus in the other, would crowd that organ against the spinal column, thereby increasing the pressure on the kidneys.

The three cases of convulsions just reported were all first pregnancies and one was a case of twins, you will remember. Lintzman suggests that the frequency of convulsions in unmarried females is not caused so much from the mental excitement arising from their unfortunate condition, as it is by the tight lacing to which they resort to conceal their shame. That obstruction of the renal veins will produce albuminuria has been repeatedly proven by ligating those vessels in animals. But,after all,the theory of renal congestion by pressure can hardly be considered as proven, for we often have albuminuria in the earlier months of pregnancy, before pressure could take place, and then we have pressure applied in the very same manner in ovarian and uterine tumors and get no albuminuria as the result.

In case No. 3 reported to-day, where the albumen ceased to be secreted with the urine and the œdema suddenly disapeared on the probable death of the fœtus, the albumen in this case could not certainly have been caused by mechanical pressure. The death of the child would not have removed the pressure if it existed, but it stopped the secretion of albumen.

With our present knowledge in regard to the etiology, I think we may sum up by saying, that no doubt in some cases, it is from a temporary Bright's Disease; or Puerperal Nephritis perhaps would be a better name. In some others it may be from mechanical pressure. In others from causes connected with the pregnancy not yet understood. As to its relations to puerperal convulsions, we know that it is a fact, that a very large proportion of cases of eclampsia occurring during pregnancy, parturition and child bed have albuminous urine. The albuminuria of course is not the cause of the convulsions. It is important only as an exponent of some abnormal renal difficulty either structural or functional; and that some pernicious elements of the urine are not properly eliminated, but remain in the circulation producing what is known as uræmic intoxication. Frericks and Lehman independently and at the same time advanced the theory that uræmia was due to some ferment that transformed the urea into carbonate of ammonia, but this I believe has never been satisfactorily proven. We only know that when the kidneys from any cause cease to properly perform their function and some of the excrementitious elements of the urine remain to pollute the blood, we get coma and convulsions as the result. But what particular element or combination of elements produces this effect, is at present not precisely known.

While a large proportion of physicians have for some time accepted the doctrine that uræmia is the principal cause of eclampsia puerperalis; there are others in this country and Europe who, although they admit the frequency of albuminuria, yet do not consider that we have sufficient proof to claim that they stand in the relation of cause and effect. On the contrary they are inclined to think that both the eclampsia and the albuminuria are due to a common cause, the exact nature of which science has not yet determined. And the reasons given for this belief are

1st That there are many cases dying from convulsions, when on post mortem, the kidneys show only a trifling, if any lesion ; surely not enough to warrant the diagnosis of Bright's disease.

2nd. That we often have albuminuria and no convulsions occurring.

3rd. That we sometimes get convulsions and fail to detect any evidence of albumen or casts in the urine.

4th. That when it does happen, that albumen exists in the urine in connection with convulsions, it often does not precede the outbreak of the attack, but makes its first appearance during the labor or after a convulsion as occurred.

Of course all convulsive movements are from excitation or irritation of the true spinal system, centric or reflex, physiologists have proven that. We also know that certain substances circulating in the blood, either generated in the system or introduced from without like strychnine, rabies etc., will produce convulsive spasms. We also know that these substances will produce a nervous irritability, that may culminate in convulsions, whenever excited by something that under ordinary circumstances would make no impression, like the falling of water in hydrophobia, or the slaming of a door etc. in strychnine poisoning. So it seems to me it is, in albuminuria in pregnancy. The toxæmia produces an excited nervous sensibility, and that this is why the act of parturition so often appears to give origin to the convulsions. But whether the toxæmia is the fault of the kidneys, or that the blood is altered by something connected with the pregnancy and causes the kidneys to secrete albumen, I will not undertake to say, that the blood is contaminated in albuminuria, even when convulsions do not occur, is shown by the fact that the life of the fœtus is always in peril and that abortion is apt to take place.

In the treatment we find the same discrepancy of opinion existing that we do in regard to the etiology and pathology. The views held in reference to the latter, are likely to modify the practice, in the former it would surely puzzle the student to know what to do if he should undertake to read up all that has been written on the subject. Whenever I am consulted in regard to an approaching confinement, I make it a rule to examine the urine. If it is albuminous and continues so, as shown by repeated examinations, even when no other abnormal symptoms exists, I carefully watch the case; for if œdema has not yet come on, it will probably not be very long before it makes its appearance. The daily loss of albumen from the circulation would soon induce a hydropic condition, and the safety of the patient will very much depend on the prophylaxis.

The skin, the bowels and the kidneys should be so far as possible made to properly perform their functions, if costive, *epsom salts* should be given. If the urine is scantily secreted, especially if dropsical, I would freely give diluents, those diuretics that act principally by their bulk. The skin should be stimulated into increased activity by warm water, or hot air baths, good nutritious food, but sparingly of meat, and plenty of fresh air.

After convulsions have occurred, venesection chloroform, cathartics and delivery are the principal remedies employed. But opinions as to the prominence that shall be given to each, or their relative importance in the treatment are widely different. There are those eminent in the profession whose position and large experience entitle them to speak with authority who would exclude venesection entirely as not only useless, but hurtful. Prof. Carl Braun in his work on uræmic convulsions of pregnancy, translated by Duncan page 149, says, "since the days of Burns and Hamilton, it has been in many places and still is the custom to find the only panacea against eclampsia in abundant general blood letting, often repeated in the course of the day. A proceding which can only be justified as little in by the present state of theoretical knowledge in regard to this disease, as it is by the great mortality of mothers and children constantly produced by this method of treatment," I believe most German physicians adopt the same views.

Prof. Elliot in his obstetrical clinic says, "I find myself resorting less frequently to this practice even, or with less confidence in the abstraction of blood in each succeeding year."

On the other hand there are those, not a few equally eminent, who teach and practice the very opposite who claim that clinical experiance and the records of practice afford abundant evidence, that in a large majority of cases, venesection is the most reliable and should stand first in importance, all others being secondary.[1]

Now who shall decide when doctors disagree? I know of no

[1] American Medical Journal, 1868.

other way than that each must decide for himself with the case before him. No doubt, a large proportion of American practitioners, while they do not discard bloodletting entirely, give it a much less prominent place in the treatment than formerly. In a strong plethoric woman I would not hesitate to bleed, and freely, if necessary, both to modify the severity of the convulsions and to prevent mischievous extravasation in the brain. Although congestion is not a cause of eclampsia, it may be a result. But as a rule the cases we have to deal with are not plethoric but the contrary. Albuminuria is decidedly an anæmic affection. The blood vessels may be full and distended, but it is watery blood. But chloroform is a remedy that is always proper to use, its efficiency and value is recognized by all. If skillfully and wisely employed, the threatened attack may sometimes be warded off, but if not they are generally rendered less severe.

Prof. Simpson was of the opinion that the benefit derived from its use was not alone due to its sedative properties, but that it to some extent renders toxæmic blood less noxious. It has been shown by chemical analysis, that its inhalation caused the secretion of sugar with the urine, the blood also probably contains it. Now a little sugar mixed with urine outside of the body prevents the ordinary change of the urine into carb. of ammonia. Therefore he was inclined to think that the sugar, resulting from the inhalation of chloroform, might be of some benefit in preventing that change in the blood. Cathartics should be given early, choosing those that act promptly and quickly, they are useful as revellents and perhaps also to eliminate some of the morbid elements of the circulation.

As to artificial delivery, when to resort to it, and when to withhold our hand and wait, is often a very grave and anxious question presenting itself to our consideration. And one that can only be decided by careful and intelligent survey of all the conditions connected with the case before us.

In Case No. 1. Mrs. H. the head of the first child was so far advanced that a few good labor pains, no doubt would have expelled it. but no pains were there to do it. It was an easy matter to apply the forceps without much disturbance of the patient, and by moderately using chloroform there was but little danger that their application would be an exciting cause of a convulsion. It also was safer for the child. In regard to the second one, it was a different question to decide, you will remember ext. ergot F. 3 I. had been given, that it had produced no pains at the end of half an hour, when a severe convulsion come on, lasting about one minute. Ordinarily I admit this would not be sufficient cause to forcibly deliver, but all of you who have had occasion to deliver a second child in twin pregnancies, by turning will remember with what facility and ease it can be done. Knowing this fact and also knowing the extreme danger to both mother and child, if many such convulsions as she had just passed through should be repeated, together with the absence of all pain, decided me to deliver; and the result was very gratifying. The mother and two babies safely delivered from their extreme peril and restored to their alarmed and anxious friends.

In the Case of Mrs. E. It was very evident that unless the pregnancy was soon terminated, she would die undelivered. There had been no labor pains, therefore we decided to interfere and deliver. The case ended favorably. Of course we cannot know, but the result would have been the same, if the operation had not been performed, but I doubt it very much. The fact alone that the albumen disappears in most all cases, very soon after the pregnancy ceases to exist, seems to me to be a sufficient reason to warrant us in delivering soon as practicable, yet I would not interfere until I was pretty sure the operation would be less likely to do harm, than the detention of the child in the uterus. With our present means at hand for producing dilitation, chloroform, warm douche, dilators, we can deliver much sooner and with less shock than formerly. In regard to the induction of labor before any convulsions have occurred, when in spite of treatment the disease progresses, when the brain, stomach, and nervous system generally indicate serious trouble, the fœtus being viable, it should be done.

But when the pregnancy is not far enough advance for the child to sustain an extra-uterine existence, and the operation is only for the interest of the mother, we would not be justified in interfering until her life was in actual danger.

